# Re-Classification of *Drosophila melanogaster* Trichoid and Intermediate Sensilla Using Fluorescence-Guided Single Sensillum Recording

**DOI:** 10.1371/journal.pone.0139675

**Published:** 2015-10-02

**Authors:** Chun-Chieh Lin, Christopher J. Potter

**Affiliations:** The Solomon H. Snyder Department of Neuroscience, Center for Sensory Biology, Johns Hopkins University School of Medicine, Baltimore, Maryland, United States of America; Center for Genomic Regulation, SPAIN

## Abstract

*Drosophila* olfactory receptor neurons are found within specialized sensory hairs on antenna and maxillary palps. The linking of odorant-induced responses to olfactory neuron activities is often accomplished via Single Sensillum Recordings (SSR), in which an electrode inserted into a single sensory hair records the neuronal activities of all the neurons housed in that sensillum. The identification of the recorded sensillum requires matching the neuronal responses with known odor-response profiles. To record from specific sensilla, or to systematically screen all sensillar types, requires repetitive and semi-random SSR experiments. Here, we validate an approach in which the GAL4/*UAS* binary expression system is used for targeting specific sensilla for recordings. We take advantage of available *OrX-Gal4* lines, in combination with recently generated strong membrane targeted GFP reporters, to guide electrophysiological recordings to GFP-labeled sensilla. We validate a full set of reagents that can be used to rapidly screen the odor-response profiles of all basiconic, intermediate, and trichoid sensilla. Fluorescence-guided SSR further revealed that two antennal trichoid sensilla types should be re-classified as intermediate sensilla. This approach provides a simple and practical addition to a proven method for investigating olfactory neurons, and can be extended by the addition of *UAS-geneX* effectors for gain-of-function or loss-of-function studies.

## Introduction

*Drosophila* olfactory receptor neurons on the third antennal segments and maxillary palps are housed in specialized porous sensory hairs called sensilla. Scanning electron and light microscopy studies have revealed that these sensory hairs can be classified based on position, size, shape, and pore densities into 4 categories: basiconic, trichoid, intermediate, and coeloconic [1]. Each sensillum contains between one to four olfactory receptor neurons (ORNs). Most ORNs express only one kind of tuning Odorant Receptor (*OrX*) in combination with the Olfactory Co-Receptor *Orco* [2]. There are ~1200 ORNs in each antenna, housed in the four sensillar categories: basiconic (ab1-ab10), trichoid (at1-at4), intermediate (ai1), and coeloconic (ac1-ac4) sensilla [3]. The maxillary palps contain an order of magnitude fewer ORNs (~120) and all of the ORNs are housed in basiconic sensilla (pb1-pb3). Direct extracellular electrophysiological recordings from sensilla have proven to be a powerful technique to understand tuning affinities of individual ORNs [4–7]. In the single sensillum recordings (SSR) technique, a recording electrode is inserted into the base of the sensillum [4]. This allows for extracellular-field potential measurements of action potentials generated by all the ORNs within a single sensillum, and provides a quantitative method to investigate olfactory responses to stimuli.

**Fig 1:**
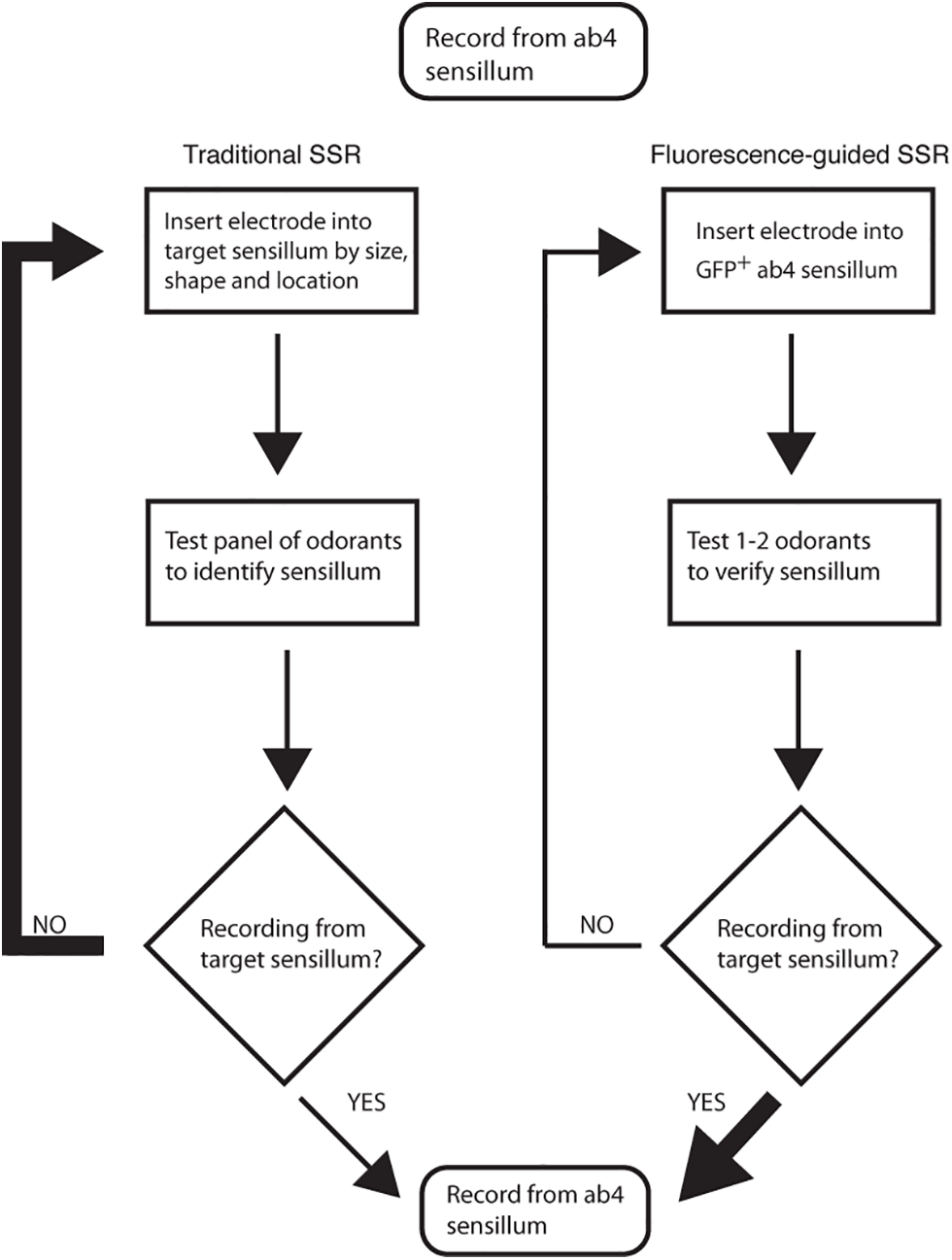
Flow chart comparing traditional SSR with Fluorescence-guided SSR. Traditional Single Sensillum Recordings (SSR) often requires screening many sensilla for odorant activities before identifying a correct target. Fluorescence-guided SSR (FgSSR) allows for GFP-labeled sensilla to be targeted directly for recordings. This increases the success rate for recording from a specific sensillum (*e*.*g*., ab4), and decreases the effort needed to record from the desired sensillum. FgSSR is also useful for targeting sparse sensilla (*e*.*g*. ab4 to ab10), sensilla with similar sizes and locations (ab6-10 with ai1-3; pb1-3), sensilla that house neurons with unknown odor-responses (*e*.*g*, ai1), or for targeting specific Odorant Receptors in unknown sensillar types. Thick “Yes/No” arrows indicate higher probabilities and thin decision arrows indicate lower probabilities.

Given the diversity of sensillar types (19 types in antenna and 3 types in maxillary palps), identification of each target sensilla for measurement of olfactory responses can be challenging and time-consuming (Fig 1). One method to circumvent this is to use the GAL4*/UAS* binary expression system [8, 9] to mis-express individual ORs in an easily identified basiconic sensilla (ab3) that houses a neuron lacking its endogenous receptor (the empty neuron system, genotype: *Δhalo/Δhalo*; *Or22a-Gal4/UAS-OrX;* [10]). This has proved to be an extremely powerful approach to define the odor profiles of many ORs [6, 7, 11]. However, several odorant receptors do not function in the empty neuron system (*e*.*g*, pheromone receptors, [12]), possibly because essential factors are lacking in the ab3A neuron or absent in the lymph surrounding the neuron [13, 14]. Therefore, direct measurement of neuronal activities in native sensilla remains the most reliable way to characterize the odor-activation profiles of individual ORNs. However, this can be particularly challenging for olfactory neurons that are sparse, or if odor-properties are not well defined.

The identification of *Drosophila* odorant receptors in the sequenced genome has allowed rapid progress in characterizing the olfactory system in this model organism [15, 16]. Using the odorant receptor promoter to drive expression of GAL4 (*OrX-Gal4*) has allowed molecular biological approaches to match odorant receptors to olfactory neurons within defined sensilla [3, 10, 17]. However, SSR approaches have yet to take full advantage of these available molecular reagents. Here, we describe Fluorescence-guided SSR (FgSSR) in which we incorporate genetic labeling techniques with electrophysiological recordings. The FgSSR approach has many advantages over traditional SSR: 1) it increases the efficiency of screening antennal and maxillary palp sensilla for odorant responses; 2) it increases the success rate for recording from any specific sensillum; and 3) it enables recording from genetically indentified but not morphologically distinct sensilla. FgSSR is useful for targeting sparse sensilla and sensilla that house neurons with unknown odor-responses. Furthermore, by comparing the sizes and shapes of the sensilla, followed by electrophysiological recordings, we identified that intermediate and trichoid sensilla have been misclassified. Here, we present validated reagents required for FgSSR and also correct a classification of intermediate and trichoid sensilla.

## Materials and Methods

### Fluorescence-guided Single Sensillum Recording (FgSSR)

**Fig 2:**
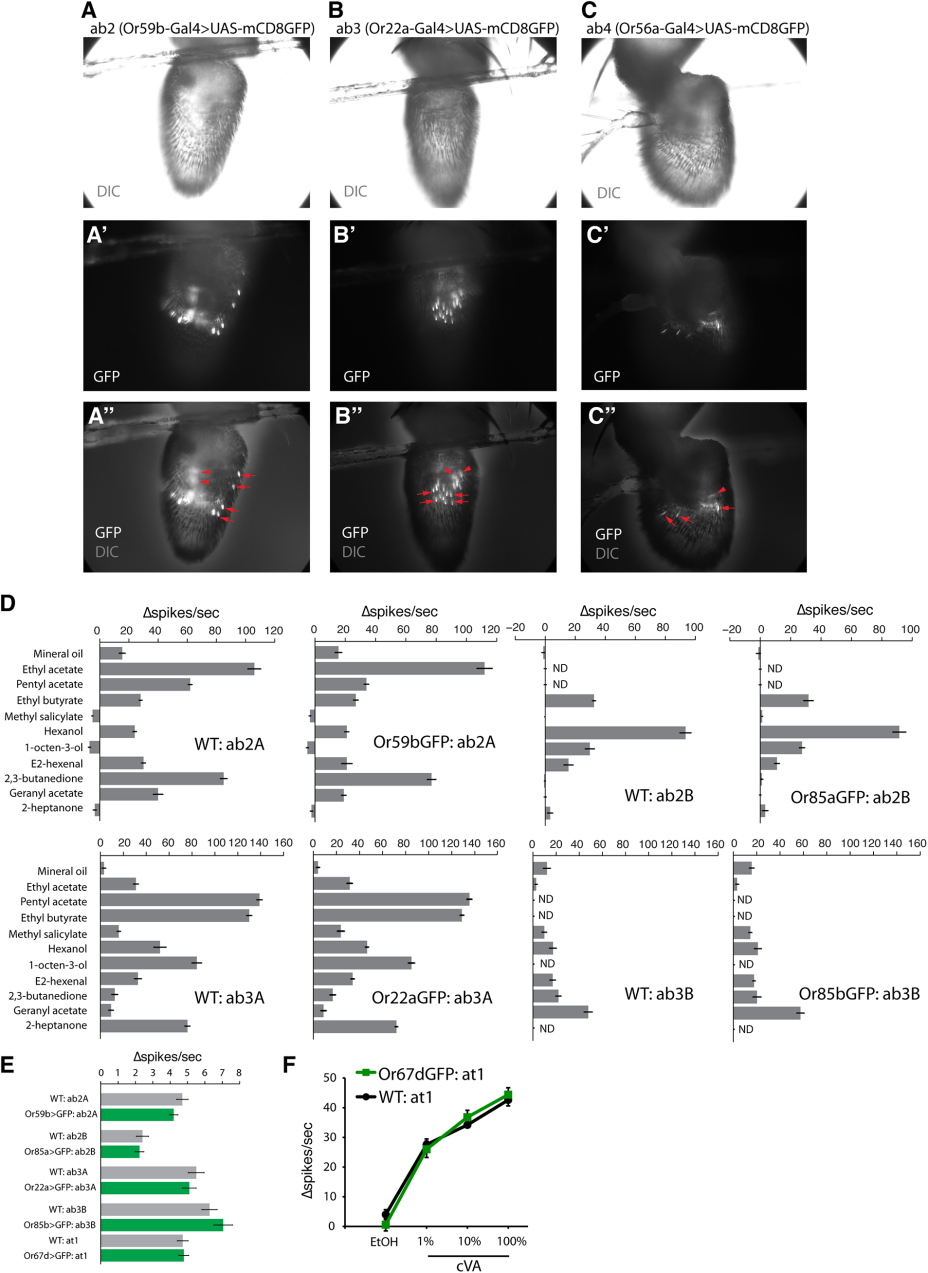
Expression of mCD8GFP in the olfactory neuron does not alter odor responses. **(A)** ab2 sensilla were labeled using *Or59b-Gal4* to drive 15x*UAS-IVS-mCD8GFP* expression. Antennae were visualized on the recording rig by differential interference contrast (DIC, top), and for GFP expression (middle), and the merged image is shown in the bottom row. **(B)** ab3 sensilla were labeled using *Or22a-Gal4* to drive 15x*UAS-IVS-mCD8GFP* expression. **(C)** ab4 sensilla were labeled using *Or56a-Gal4* to drive 15x*UAS-mCD8GFP* expression. In (**A-C**), arrowheads point to example cell body labeling, and arrows point to example sensillum labeling. **(D)** Comparing the SSR odor response profiles of wild-type (*WT*) and GFP-expressing neurons in ab2 and ab3 sensilla. The odor response profiles to 10 standard odorants plus mineral oil were examined for unlabeled *WT* and FgSSR-targeted ab2A, ab2B, ab3A, and ab3B neurons. Responses to all odorants were similar, with the exception of ab2A response to pentyl acetate, which was decreased in the FgSSR experiment (n = 4–6 for each recording). **(E)** The spontaneous activities of WT and GFP-labeled ab2A (Or59b), ab2B (Or85a), ab3A (Or22a), ab3B (Or85b), and at1 (Or67d) showed no significant differences (n = 9 for each recording). **(F)** Comparing the SSR odor response profiles of wild-type (*WT*) and GFP-expressing at1 Or67d+ neurons (*Or67d-Gal4/15xUAS-mCD8GFP*) to the pheromone ligand (cVA) at different pheromone concentrations (n = 5). Error bars indicate ± s.e.m. throughout.

**Fig 3:**
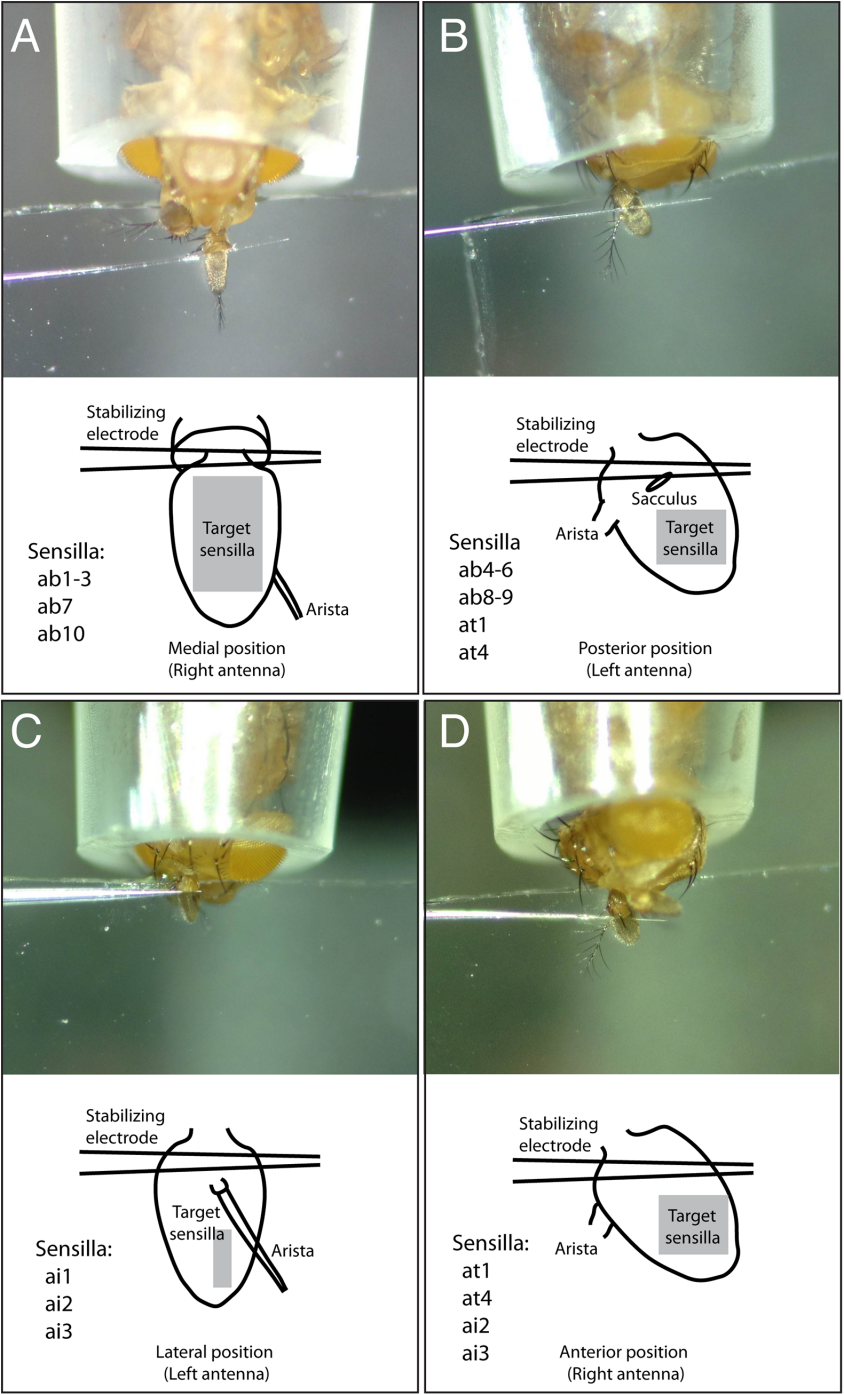
Suggested mounting positions and target zones. Shown are images of mounted flies (top) and cartoons of prepared antenna (below). (A) A medial mounting position exposes ab1-3, ab7 and ab10 basiconic sensilla. (B) A posterior mounting position exposes ab4-6 and ab8-9 basiconic sensilla, as well as at1 and at4 trichoid sensilla types. (C) A lateral position exposes ai1-3 intermediate sensilla. (D) An anterior mounting position exposes at1, at4, ai2 and ai3 sensilla.

Sensilla of targeted ORNs were identified using 10x and 50x objectives with an optovar 1.6x attachment (Zeiss, EC Epiplan-Neofluar 10x, LC EC Epiplan-Neofluar 50x and Optovar Module 1.6x P&C ACR) on a Zeiss AxioExaminer D1 compound microscope, using a light source and eGFP filter cube (FL Filter Set 38 HE GFP shift free). Green fluorescence signals in flies were visualized from *OrX-Gal4* and *10xUAS-IVS-mCD8GFP* (Bloomington Stock #32186; for the *OrX-Gal4* on Chr. II) or *15xUAS-IVS-mCD8GFP* (Bloomington Stock #32193; for the *OrX-Gal4* on Chr. III). The representative images shown in Fig 2A–2C and S1 Fig were taken on the recording rig. The suggested mounting positions of antenna are shown in Fig 3. The electrode was filled with Beadle-Ephrussi ringers solution (7.5g of NaCl+0.35g of KCl+0.279g of CaCl_2_-2H_2_O in 1L of H_2_O). Extracellular activity was recorded by inserting a glass electrode into the base of the sensillum of 4–8 day-old flies. Signals were amplified 100X (USB-IDAC System; Syntech, Hilversum, The Netherlands), inputted into a computer via a 16-bit analog-digital converter and analyzed off-line with AUTOSPIKE software (USB-IDAC System; Syntech). The low cutoff filter setting was 50Hz, and the high cutoff was 5kHz. Stimuli consisted of 1000 ms air pulses passed over odorant sources. The Δspikes/second was obtained by counting the spikes in a 1000ms window from 500 ms after odor stimuli were triggered, subtracting the spikes in a 1000ms window prior to stimulation. 10 standard odors for identification of sensillar types are acquired at highest purity and listed as follows: Ethyl acetate (Sigma #270989), Pentyl acetate (Sigma #109584), Ethyl butyrate (Sigma #W242705), Methyl salicylate (Sigma #76631), Hexanol (Sigma #471402), 1-octen-3-ol (Sigma #68225), E2-hexenal (Sigma #W256005), 2,3-butanedione (Sigma #B85307), Geranyl acetate (Sigma #45896), 2-heptanone (Sigma #537683), 11-cis vaccenyl acetate (50mg in 1ml ethanol, Cayman Chemical Company #0424297–6). The odors except cVA were diluted in mineral oil (Sigma #330779) at 1:100 and 30 μl was used for stimulation. Odors were delivered to the antenna as previously described [4, 6, 10]. Stimuli were delivered by placing the tip of an odor Pasteur pipette through a hole in a pipette (Denville Scientific Inc, 10ml pipette) that carried a purified continuous air stream (8.3 ml/s) directed at the antenna. A solenoid valve (Syntech) diverted delivery of a 1 s pulse of charcoal-filtered air (5 ml/s) to a Pasteur pipette containing odorant dissolved onto filter paper. Fresh odorant pipettes were used after no more than 3 odor presentations.

### Channel Rhodopsin Activation

Newly eclosed flies (age < 1 day old) were transferred to fly vials containing 0.4 mM of all-trans retinal (Sigma-Aldrich # R2500, dissolved in pure ethanol or DMSO) with fly food. The vials were kept in the dark for at least 4 days before experiments. 627nm LED light source (1-up LED Lighting Kit, PART #: ALK-1UP-EH-KIT) powered by an Arduino Uno (www.arduino.cc/en/Main/ArduinoBoardUno) was used to activate CsChrimson (Bloomington Stock # 55135 and Bloomington Stock # 55136). The light intensity was adjusted to 1.13 W/m^2^ by setting to 2 V supply using a custom Arduino program with a distance between the LED lens and fly antenna at 20 cm.

### Differential Interference Contrast (DIC) and Confocal Imaging

**Fig 4:**
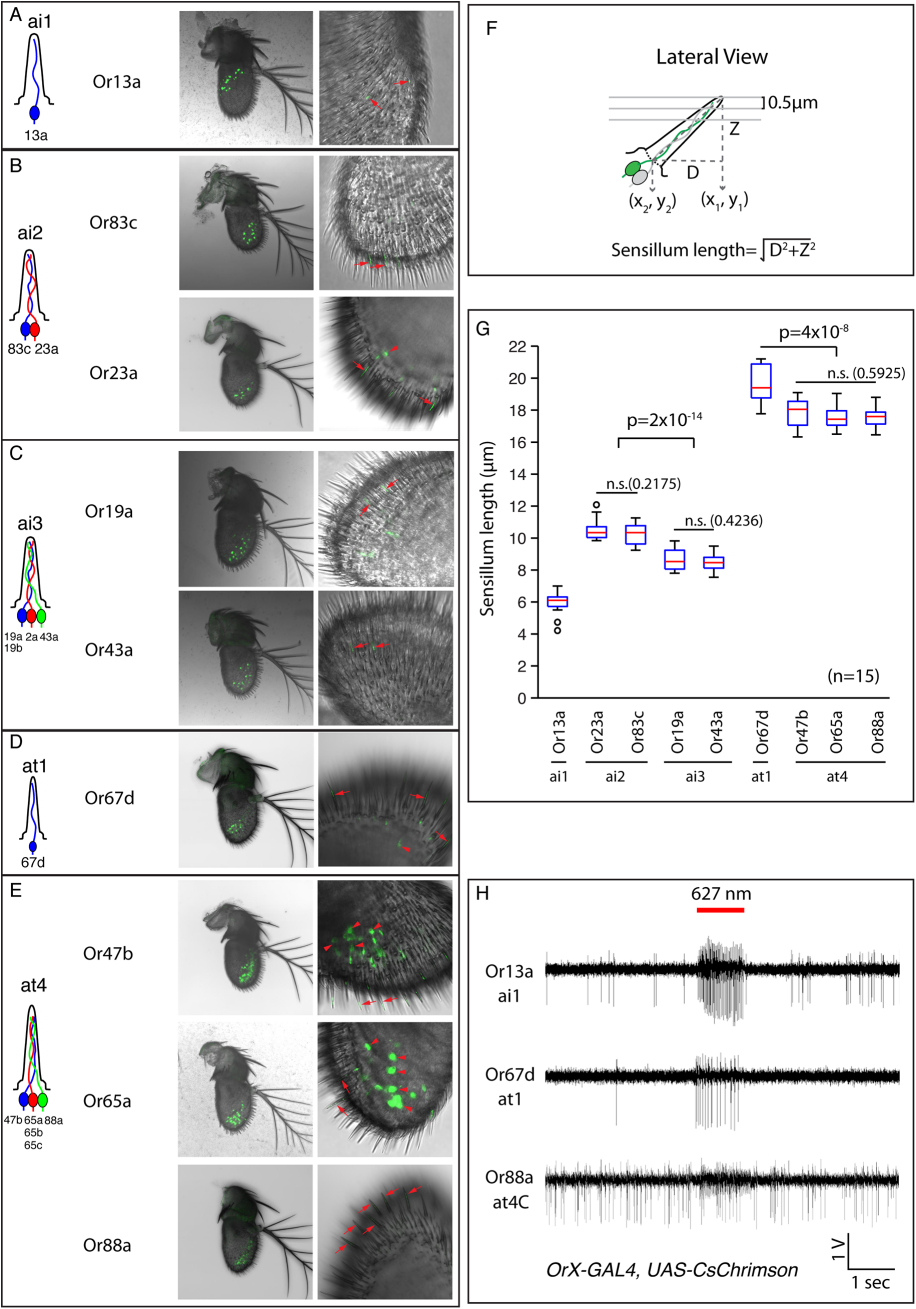
Simultaneous labeling and visualization of intermediate and trichoid sensilla by FgSSR. GFP-labeling of sensilla allows for accurate classification of sensillar types. **(A-E)** *OrX-Gal4* with *15xUAS-IVS-mCD8GFP* clearly labels a specific olfactory neuron and sensilla. Middle panels show confocal DIC images of the antenna overlaid with the GFP signal, and right panels shows a higher magnification of the GFP-labelled sensilla. Arrowheads point to example cell body labeling, and arrows point to example sensillum labeling. **(A)** Ai1 sensilla identified by *Or13a-Gal4*. **(B)** Ai2 sensilla identified by *Or23a-Gal4* and *Or83c-Gal4*. **(C)**. Ai3 sensilla identified by *Or19a-Gal4* and *Or43a-Gal4*. **(D)** At1 sensilla identified by *Or67d-Gal4*. **(E)** At4 sensilla identified by *Or47b-Gal4*, *Or65a-Gal4*, and O*r88a-Gal4*. **(F)** Cartoon showing how measurement of sensillar length is performed. **(G)** Characteristic lengths of intermediate and trichoid sensilla. *p-*values are as shown. t-test, n = 15 per genotype. Error bars indicate ± 2.5 s.e.m. throughout. Data points not within this range are plotted as circles. **(H)** FgSSR combined with *20xUAS-IVS-CsChrimson* allows light-guided stimulation of olfactory neurons. This confirms at4C neurons express Or88a.

The images shown in Fig 4A–4E were Z stacks of 3–4 DIC and confocal section images. Third antennal segments of 4–6 day-old flies were dissected in PBT solution (1X PBS with 0.3% Triton X-100) and fixed in 4% PFA (PBS: 70%, PBT: 10%, PFA (20%): 20%) for 15 minutes. Fixed antenna were washed with PBT for 20 minutes and mounted in PBT for imaging. DIC images and endogenous fluorescence were taken on a LSM 700 Confocal Microscope (Zeiss). Specimens were imaged fresh or no longer than 2 days after dissection since endogenous fluorescence signals would decay over time.

### Calculation of Sensillar Length

A target sensillum was first identified using *OrX-Gal4* driven GFP fluorescence. The positions of the tip and base of the antenna were then recorded using DIC images. Briefly, the D length (Fig 3F) was calculated using the x and y positions between tip and base. Assuming tip position to be (x1, y1) and base to be (x2, y2), the distance would be [(x1-x2)^2^+(y1-y2)^2^]^1/2^. Z distance was decided by the number of fixed width confocal sections (thickness is 0.5 μm in this study). With D and Z, the sensillum length was calculated as [D2+Z2]^1/2^.

## Results and Discussion

### Fluorescence-guided SSR: coupling electrophysiological recordings with fluorescence-targeted olfactory neurons

Traditional single sensillum recordings (SSR) rely on the sizes, shapes, locations and neuronal odor responses (typically to a panel of up to 10 standard odors) to correctly identify target sensilla. This approach is often time-consuming and requires extensive training (Fig 1).

The introduction of binary expression systems into the *Drosophila* toolbox has revolutionized transgenic experimental approaches [9, 18–20]. Odorant receptor-specific promoters can be used to identify olfactory neurons by driving fluorescent protein expression in an olfactory neuron of interest (*e*.*g*. O*rX-Gal4*, *UAS-mCD8GFP*). This approach was used successfully for targeting ab3 sensilla for SSR (*Or22a-Gal4*, *5xUAS-mCD8GFP*) [10]. However, traditional reporter lines, such as *5xUAS-mCD8GFP*, do not typically label sensilla with sufficient strength to allow sensillum identification [10]. As a result, the use of the GAL4/*UAS* system as a companion to SSR has not been widely adopted. Recent improvements to the *UAS-mCD8GFP* reporter, in which the number of GAL4 binding sites (upstream activation sequence, *UAS*) were increased to 10, 15, 20 and 40 copies, allowed for significant increases in labeling strength [21]. We reasoned that these new reporters might now allow for sufficient labeling of olfactory sensilla by existing *OrX-Gal4* lines.

We acquired all available *OrX-Gal4* lines from the Bloomington Stock Center, and validated which lines, when combined with *10xUAS-IVS-mCD8GFP* (on Chromosome 2) or *15xUAS-IVS-mCD8GFP* (on Chromosome 3), drove sufficient expression in only a single type of olfactory neuron and were still strong enough to allow identification of sensilla when viewed on a SSR compound microscope setup (Table 1; Material and Methods). This should not only significantly shorten the time span needed to identify small sensilla (*e*.*g*. ab4-ab10, pb1-3, ai1-3) but also provide a direct method to record from genetically identifiable sensilla (Fig 1). We call this method Fluorescence-guided Single Sensillum Recording (FgSSR).

**Table 1:** Validated *OrX-Gal4* reagents for FgSSR.

Sensilla (Neuron)	Receptors	Bloomington Stocks for Validated transgenic lines for FgSSR^a^	Suggested mounting positions
ab1	Or92a, Or42b, Gr21a, Or10a	No need^b^	Medial
ab2 (A)	Or59b	23897	Medial
ab2 (B)	Or85a	24461	Medial
ab3 (A)	Or22a	9951	Medial
ab3 (B)	Or85b	23911	Medial
ab4 (A)	Or7a	KI	Posterior
ab4 (B)	Or56a	23896	Posterior
ab5 (A)	Or82a	23126	Posterior
ab5 (B)	Or47a	9982	Posterior
ab6 (B)	Or49b	9986	Posterior
ab7 (A)	Or98a	23141	Medial
ab7 (B)	Or67c	23905	Medial
ab8 (A)	Or43b	23894	Posterior
ab9 (B)	Or67b	9995	Posterior
ab10 (B?)	Or67a	23904	Medial
at1 (A)	Or67d	KI [22]	Posterior or Anterior
at4 (A)	Or47b	9983	Posterior or Anterior
at4 (B)	Or65a	9994	Posterior or Anterior
at4 (C)	Or88a	23138	Posterior or Anterior
ai1 (A)	Or13a	23886	Posterior
ai2 (A)	Or83c	23131	Lateral
ai2 (B)	Or23a	9956	Lateral
ai3 (A)	Or19a	23887	Lateral
ai3 (C)	Or43a	9974	Lateral
pb1^c^ (A)	Or42a	9969	Posterior
pb1^c^ (B)	Or71a	23122	Posterior
pb2^c^ (A)	Or33c	9966	Posterior
pb3^c^ (A)	Or59c	23899	Anterior
pb3^c^ (B)	Or85d	24148	Anterior

^**a**^ *OrX-Gal4* lines on II were recombined with *10xUAS-IVS-mCD8GFP* (Chr II attP40; BS#32186). *OrX-Gal4* lines on III were recombined with *15xUAS-IVS-mCD8GFP* (Chr III attP2; BS#32193).

^**b**^ ab1 sensilla are the only large basiconic sensilla that contain 4 neurons and thus very easily identified.

^**c**^ Mounting positions of pb sensilla are less strict since they are more uniformly distributed. Homozygous lines (*OrX-Gal4*, *UAS-mCD8GFP*/*OrX-Gal4*, *UAS-mCD8GFP*) are not recommended in maxillary palp ORNs as thinner cuticles cause high fluorescence signals from the cell bodies. The thicker cuticle of the antenna greatly reduces visible cell body fluorescence and allows for the use of homozygous reporter lines when identifying antenna sensilla. Homozygous reporter lines are especially useful when targeting trichoid sensillum.

### Fluorescence protein expression does not interfere with olfactory activation patterns

Labeling of olfactory neurons within sensilla required high expression of membrane-targeted GFP. Since olfactory neurons require the proper functioning of membrane-bound receptors, we first questioned if membrane-targeted GFP expression in ORNs might interfere with the endogenous dynamics of ligand–receptor activation. A panel of 10 standard odorants for sensilla type identification [5, 6, 10] was applied to stimulate wild-type (*WT*) and GFP-labeled ab2A, ab2B, ab3A and ab3B neurons. There were no significant differences between the firing patterns of *WT* and GFP-labeled ORNs (Fig 2D). Furthermore, the basal firing rates of each GFP-labeled ORN were similar to those of *WT* (Fig 2E). Similarly, the stimulation of wild-type and GFP-labeled at1 neurons using the pheromone cis-vaccenyl acetate (cVA) demonstrated no differences (Fig 2F). Thus, using ORNs in the ab2, ab3, and at1 sensilla as a proof-of-principle, these results indicated that the high levels of membrane-bound fluorescence protein needed for sufficient labeling did not affect endogenous olfactory receptor activities.

We comprehensively examined and combined available *OrX-Gal4* lines with *10xUAS* or *15xUAS-IVS-mCD8GFP* lines to cover all known basiconic, intermediate, and trichoid sensillar types (ab1-10, at1-2, ai1-3 and pb1-3; Table 1). For each sensillar type, at least one *OrX-Gal4* line was examined by brain dissection to ensure specific and correct targeting to the reported glomerulus in the antennal lobes [3, 17]. We suggest specific *OrX-Gal4* lines to use for targeting corresponding sensilla (Table 1). We also determined optimal mounting positions and the targeted regions for single sensillum recording (Fig 3 grey zones). A medial position exposed most basiconic sensilla (ab1-3, ab7 and ab10). A posterior mounting position covered basiconic (ab4-6 and ab8-9) and trichoid (at1 and at4) sensillar types. A lateral position exposed intermediate (ai1-3) sensilla. An anterior position was not frequently used because it is challenging to stabilize, but would expose at1, at4, ai2 and ai3 sensilla. Note that these positions are suggested positions to facilitate recording but were not the only positions in which the sensilla were located. For instance, ab4 sensilla were also detected in a medial mounting position but were more challenging to record in this position compared to a posterior position.

When performing SSR experiments, some sensilla are easily distinguished based on their olfactory neuron number, size, shape, and relative location on the antenna (*e*.*g*., ab1, ab2, and ab3), and do not necessarily require FgSSR (especially for ab1, which is the only large basiconic containing 4 neurons). However, small basiconic sensilla (ab4-ab10, pb1-pb3) and intermediate sensilla (ai1-ai3) are extremely difficult to distinguish on an SSR scope based on morphology alone. FgSSR allows each sensillum to be un-ambiguously identified.

The utility of FgSSR can be extended by including additional *UAS-geneX* effectors that might affect neuronal function. This could be used for loss-of-function studies (*e*.*g*., expression of a *UAS-RNAi* construct [23, 24]) or gain-of-function studies. As a proof-of-principle of the second approach, we simultaneously expressed mCD8GFP and CsChrimson in genetically specified neurons and identified the endowed light-activated responses of recorded olfactory neurons. CsChrimson is a Channel Rhodopsin modified to be activated by red-shifted light [25]. We were able to identify by GFP and verify the light-evoked neuronal activities generated in specific ORNs (Fig 4H). Or13a, which has no known odor ligand, was previously assigned into an intermediate sensillum containing only one neuron [10]. We verified this result by showing that the Or13a+ sensillum expressing CsChrimson contained only a single amplitude spike that was photo-activated by red light (genotype: *Or13a-Gal4*, *15xUAS-IVS-mCD8GFP; 20xUAS-IVS-CsChrimson*) (Fig 4H). As further validation, we expressed CsChrimson and mCD8GFP using *Or67d-Gal4* and *Or88a-Gal4* and verified the olfactory neuron identities as at1 and at4C respectively. The examples also demonstrate the use of CsChrimson to verify the identity of an *OrX*-expressing neuron within a sensillum without the need for a specific odorant. These examples further demonstrate that this approach can be used for spike sorting by linking *OrX* expression to an olfactory neuron identity (*e*.*g*., Or88a expresses in the ‘C” neuron in at4).

### Re-classification of trichoid sensilla

Trichoid sensilla are the largest cuticular apparatus in the third segment of antenna, ranging from 18 to 22 μm, and have the thickest sensillar wall (350–450 nm) [1]. Trichoid ORNs are generally tuned to detect pheromones [26] and reside in 4 different subtypes of trichoid sensilla—at1 (Or67d), at2 (Or23a, Or83c), at3 (Or2a, Or19a/b, Or43a) and at4 (Or47b, Or65a/b/c, Or88a) (Fig 4) [3, 17, 22]. The odorants that activate each trichoid ORN remain to be identified. So far, it has been found that Or67d is activated by the male pheromone cVA [22, 27], Or47b is activated by the pheromone methyl laurate and Or88a is activated by the pheromones methyl laurate and methyl myristate [28]. In contrast, the identified ligands for at2 and at3 neurons are not pheromones (Or83c responds to farnesol [12] and Or19a to citrus food odors [29]). This raised the question if at2 and at3 are indeed a category of trichoid sensilla.

**Fig 5:**
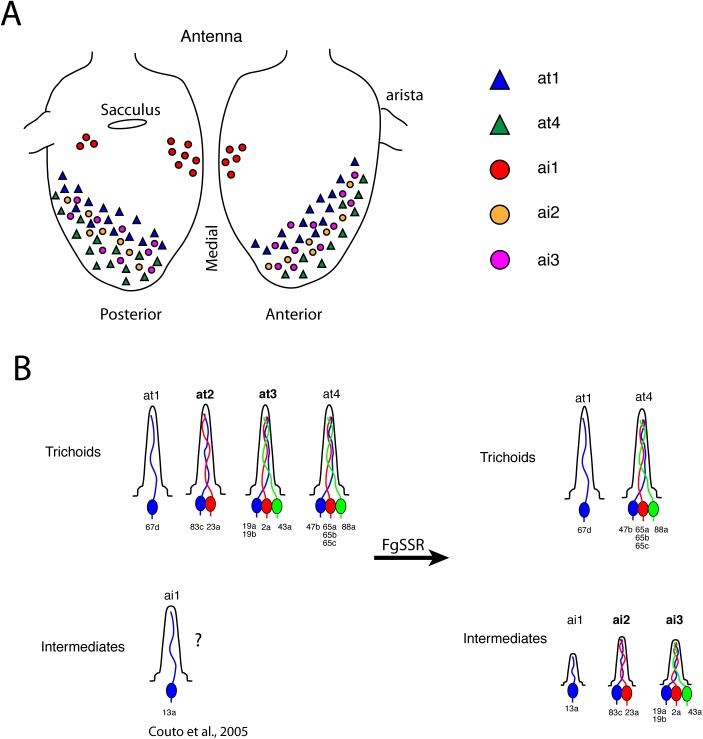
Re-classification of *Drosophila melanogaster* intermediate and trichoid sensilla. **(A)** Schematic for the location of trichoid and intermediate sensilla on the antennae. **(B**) FgSSR analyses re-classified two trichoid sensilla (names bolded) as intermediate sensilla. Intermediate sensilla identification numbers are correlated to the number of housed neurons: ai1 contains one neuron, ai2 contains two neurons, and ai3 contains three neurons.

During our validation of FgSSR, we found that some ORNs previously classified as “trichoid” [3] were not housed in what appeared to be trichoid sensilla. Instead, they were positioned in significantly shorter sensilla with thinner cuticles that are similar to intermediate sensilla [1]. With DIC combined with confocal images as the measurement of sensillar lengths (Fig 4A–4E), we found that these other sensilla were significantly shorter than trichoid sensilla, ranging from 10.38 ± 0.17μm for at2 and 8.49 ± 0.17 μm for at3 (as compared to traditional trichoid sensilla: 19.56 ± 0.31 μm for at1 and 17.7 ± 0.22 μm for at4) (Fig 4F and 4G). As the significant length differences between at1/at4 and at2/at3 likely underlied different categories of sensilla, we reclassified at2 and at3 into ai2 and ai3 respectively (Fig 5). To avoid confusion between past and future SSR studies, we did not rename at4. The 4 types of sensilla were distributed in the distolateral region on the antenna, with at1 in the proximal region, at4 at the distal region and intermixed ai2 and ai3 in between at1 and at4 (Fig 5A).

## Conclusions

The FgSSR approach described here can be used for rapid and systematic screening of odor responses in all basiconic, intermediate, and trichoid olfactory receptor neurons. Traditional SSR screens require a known odor-response profile to guide sensillar identification, and may easily miss sensilla that are rare, or whose response profiles can be confounded. FgSSR, in contrast, provides an un-biased approach, and does not require dependence on an odor-profile for identification, which can prove particularly useful for pheromone receptor neurons whose odorants are unknown or are difficult to acquire (*e*.*g*., ai1A Or13a+). In addition, since all sensilla of the same type are labeled during a single experiment, independent recordings from a sensilla type can be achieved more quickly and easily.

Our experiments have led to a re-classification of sensillar types by expanding the number of intermediate sensilla at the expense of trichoid sensilla (Fig 5; also see [12]). We suggest that there are two types of trichoid sensilla (at1 and at4), and three types of intermediate sensilla (ai1, ai2, ai3). Our classification scheme, in which the intermediate sensilla identification number also reflects the number of housed neurons, will increase the ease by which these sensilla are classified across different research groups.

## Supporting Information

S1 Fig Fluorescence-guided images of additional basiconic sensilla. Ab5-ab10 sensilla were labeled using *OrX-Gal4* to drive *UAS-mCD8GFP* (*10xUAS* or *15xUAS*) expression. Antennae were visualized by differential interference contrast (DIC; top), and for GFP expression (middle) on the recording rig. The merged image is shown below.
(TIF)
