# Supplementary figures and images for: Re-Classification of Drosophila melanogaster Trichoid and Intermediate Sensilla Using Fluorescence-Guided Single Sensillum Recording
Source: PLoS One. 2015 Oct 2;10(10):e0139675. doi: 10.1371/journal.pone.0139675 (PMC4592000; doi:10.1371/journal.pone.0139675)

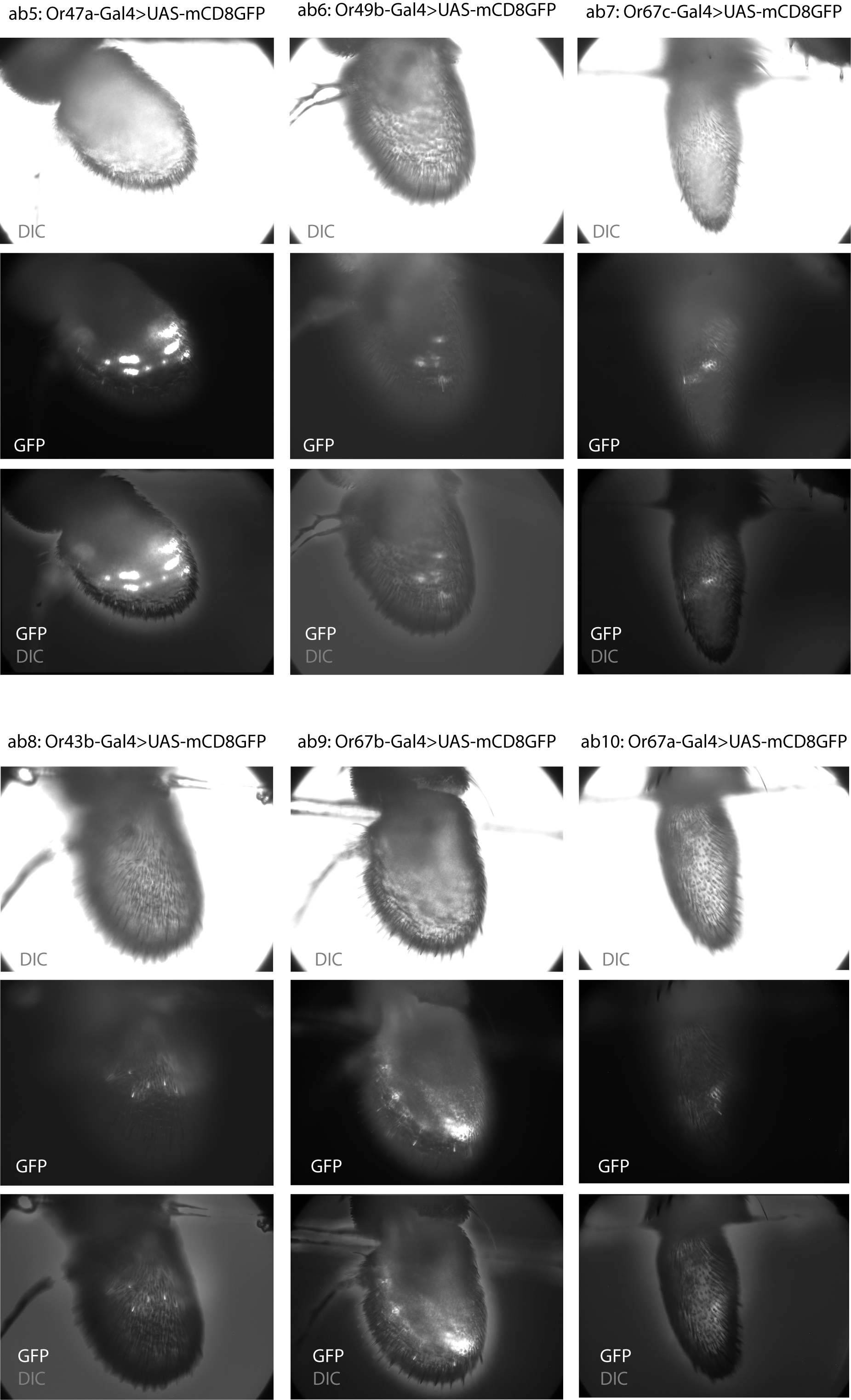

Supplement: S1 Fig — Ab5-ab10 sensilla were labeled using OrX-Gal4 to drive UAS-mCD8GFP (10xUAS or 15xUAS) expression. Antennae were visualized by differential interference contrast (DIC; top), and for GFP expression (middle) on the recording rig. The merged image is shown below. (TIF) [file pone.0139675.s001.tif]
